# Current controversies and irresolvable disagreement: the case of Vincent Lambert and the role of ‘dissensus’

**DOI:** 10.1136/medethics-2019-105622

**Published:** 2019-08-08

**Authors:** Dominic Wilkinson, Julian Savulescu

**Affiliations:** 1 Oxford Uehiro Centre for Practical Ethics, University of Oxford, Oxford, UK; 2 John Radcliffe Hospital, Oxford, UK; 3 Murdoch Children’s Research Institute, Melbourne, Australia

**Keywords:** clinical ethics, disability, resource allocation, prolongation of life and euthanasia, neuroethics

## Abstract

Controversial cases in medical ethics are, by their very nature, divisive. There are disagreements that revolve around questions of fact or of value. Ethical debate may help in resolving those disagreements. However, sometimes in such cases, there are opposing reasonable views arising from deep-seated differences in ethical values. It is unclear that agreement and consensus will ever be possible. In this paper, we discuss the recent controversial case of Vincent Lambert, a French man, diagnosed with a vegetative state, for whom there were multiple court hearings over a number of years. Both family and health professionals were divided about whether artificial nutrition and hydration should be withdrawn and Lambert allowed to die. We apply a ‘dissensus’ approach to his case and argue that the ethical issue most in need of scrutiny (resource allocation) is different from the one that was the focus of attention.

The case of Frenchman Vincent Lambert is the latest controversial example of disputed treatment for adult patients with profound brain injury.^1 2^ Lambert was seriously injured in a motorcycle accident in 2008, and subsequently diagnosed to be in a vegetative state (VS). His wife, who was his legal guardian, wished artificial nutrition and hydration to be stopped and Vincent allowed to die. His parents were strongly opposed to this. His case was heard multiple times in French and European courts.^3–5^ In June 2014, the French Conseil d’Etat (Supreme Administrative Court) concluded that Lambert’s doctors’ decision to withdraw artificial nutrition and hydration was lawful.^6^ The following year, the European Court of Human Rights found that this decision did not breach Article 2 (the right to life) of the European Convention.^6^ Treatment was withdrawn on 20 May 2019, but was reinstated within 24 hours when Lambert’s parents succeeded in a last minute legal appeal. The appeal court ordered treatment to recommence pending a review by a UN Committee on the Rights of Persons with Disabilities (UNCRPD).^1 2^ Then, on 28 June, the French Cour de Cassation overturned the lower court ruling,^7^ allowing Lambert’s doctors to reinstitute their plan to sedate Lambert and withdraw treatment. Vincent Lambert died on 11th July, nine days after artificial nutrition was ceased.

The Lambert case has obvious parallels with the US case of Terri Schiavo, but there have been other similar high-profile cases, over more than 40 years (box 1).

Box 1 **Controversial court cases relating to withdrawal of treatment from adults with profound brain injury (the dates below correspond to the dates of court hearings relating to life-prolonging treatment)**

- Vincent Lambert (France 2014–2019).
- Elena Englaro (Italy 1999–2008).
- Terri Schiavo (USA 1998–2005).
- Tony Bland (UK 1993).
- Nancy Cruzan (USA 1988–1990).
- Karen Ann Quinlan (USA 1975–1976).

There are contrasting immediate responses to cases like that of Lambert. Some people read of his case and react with horror at the idea of being kept alive indefinitely in a state of complete dependence and lack of awareness. Others have the opposite response. They respond with outrage at the idea of stopping feeding and allowing to die a profoundly disabled man, who does not appear to be suffering.^i^ A survey of the general public, published in 2014, indicated that 40% supported withdrawal of treatment from patients in VS, while 40% were unsure and 18% were opposed.^8^ A recent international survey of the lay public found that 49% agreed or strongly agreed with withdrawal of treatment, while 9% disagreed.^9^ In the same survey, two-thirds of those surveyed agreed or strongly agreed with withdrawal of treatment if they themselves were in a VS.^9^

The recurrent nature of debate over treatment for patients in a VS suggests that it is unlikely that our communities will reach a common view on this contentious ethical issue. There then becomes a question about how to deal with the issue of ethical disagreement. Recently, following another controversial example of disputed treatment (the Charlie Gard case), we proposed a ‘dissensus’ framework that might be applied in such cases (box 2).^10^ In this paper, we examine the implications of dissensus for debates about treatment for patients like Vincent Lambert. Our aim is not to settle those debates here. Rather, this analysis suggests that the most important question to address may be different from the one that is most commonly the focus of ethical attention.

Box 2 **The dissensus framework**

**Background**

It is valuable to seek agreement and consensus about important issues in medical ethics. However, in situations of disagreement based on core ethical values, consensus may not be possible. In those situations, we must make decisions despite disagreement.

**Dissensus:**

1. Separate out questions of benefit or harm for the individual patient (best interests) from questions of benefit or harm to the wider community (resource allocation/distributive justice).
2. *Individual benefit/harm*: identify the range of reasonable disagreement about options. There is no need to reach collective agreement about individual benefit. Offer options that are desired by the patient or surrogate and offer possible benefit (outweighing harm) within the range of reasonable disagreement.
3. *Collective benefit/harm*: identify reasonable disagreement about allocating resources. There is a need to reach collective agreement about options that represent a limited resource, and may harm others if provided (or reduce available options to others). Provide treatments that are judged of probable net benefit in the light of scarce resources. Allow individuals to self-fund treatments if they are of possible benefit, but cannot be provided within a publicly funded healthcare system.

The first stage in the dissensus approach is to separate out questions of individual benefit/harm from questions of collective benefit/harm. As will become clear, the ethical approach to disagreement is different in these two different situations.

## Is continuation or withdrawal of treatment best for the patient?

There are contrasting ethical arguments about treatment for patients in a VS.

### In favour of withdrawal

Some pointed to the lack of benefit for Vincent Lambert in continuing existence in a state of unconsciousness. Because he lacked any conscious interests, it was not in his best interest to keep him alive.^11^ If treatment did not confer any positive benefit to Vincent, it may be regarded as futile, or pointless.^12^ French legislation on patient’s rights and end-of-life issues specifically direct doctors not to continue treatment with ‘unreasonable obstinacy’,^3^ this includes treatment ‘n’ont d’autre effet que le seul maintien artificiel de la vie’ (having no effect other than to maintain life artificially).^13^

In Vincent Lambert’s case, his wife maintained that while he did not have a written advance directive, he had repeatedly expressed his wish not to be kept alive in a highly dependent state. The Conseil d’Etat ruling had noted

It is apparent …from the testimony of Mrs Rachel Lambert, that she and her husband, both nurses, had often discussed their respective professional experiences… and that Mr Lambert had on several such occasions clearly voiced the wish not to be kept alive artificially if he were to find himself in a highly dependent state. The tenor of those remarks, reported by Mrs Rachel Lambert in precise detail and with the corresponding dates, was confirmed by one of Mr Lambert’s brothers^6^

If this is correct, continued treatment violated his prior autonomy, and seemed to frustrate Lambert’s right to have a say in his medical care and his right not to have his life prolonged in a state that he would have regarded as deeply undesirable. It may be thought to deprive Lambert of his right to die with dignity,^14 15^ and to discriminate against him since, were he not so profoundly disabled, he would have been able to refuse treatment.

### Against withdrawal

However, it is not clear that artificial nutrition and hydration was ‘futile’ for Vincent Lambert.^16^ Such treatment had manifestly succeeded in the goals of providing nourishment and in keeping him alive over the last 11 years. It may be that some judge the quality of his life to be too low to warrant keeping him alive, but others, notably Lambert’s parents, disagreed. Whether it is ‘reasonably’ or ‘unreasonably’ obstinate to sustain life in this state is an ethical, not a scientific, question. While some, as noted above, would have found his continued treatment undignified, dignity is a deeply contested concept in bioethics.^17 18^ Others argued that Lambert’s essential human dignity remained despite his profound disability, and that his life should have been protected ‘until its natural end’.^19^

The UNCRPD was due to hear a petition relating to the Lambert case. This committee might have had concern for the rights of the severely disabled to receive life-prolonging medical treatment. The decision to withhold treatment from Lambert was directly related to the extent of his brain injury. If he were less severely disabled, artificial nutrition and hydration would potentially bring benefits other than to maintain life artificially; there would not be a question of ‘unreasonable obstinacy’. As a consequence, the UNCRPD might have felt that the Conseil d’Etat decision represented discriminatory denial of healthcare on the basis of disability (and was thus arguably contrary to Article 25 of the UN Convention on the Rights of Persons with Disabilities).^20^

However, there is a potentially competing right. Article 12 of the UN Convention emphasises the importance of advance planning, and of respecting the wishes and preferences of disabled persons (including their prior expressed preferences).^21^ If the decision, by Lambert’s doctors, was based on an accurate assessment of his individual situation, of his best interests and his wishes, it arguably did not in any way represent unjust discrimination. Furthermore, if it is accepted that Vincent had ‘no interests’ because he was permanently unconscious (as was concluded in the case of Tony Bland),^11^ it is not clear that he could be discriminated against.

### Dissensus

It can be useful to debate such questions, yet the long history of similar cases points clearly to the ongoing challenge of reaching a common view on these issues. Quite simply, we will never all agree on what should happen.

Our societies are increasingly diverse. In societies like France, the UK, the USA and many others, we have to accept and tolerate that people have a range of different values. That acceptance and tolerance means that we should allow people to live their lives based on their own ethical views and values (as long as they do not harm others). It is perfectly acceptable for people to express their views about situations like Vincent Lambert. However, it would not be acceptable to impose those views onto Vincent’s life.

Sometimes, in the face of a lack of consensus, it can be tempting to avoid making decisions. However, in situations like this, there is no neutral alternative. Continuing treatment is, itself, a decision.^ii^

The best ethical response to reasonable disagreement in cases like that of Vincent Lambert is to make decisions based on his values and his wishes. If Vincent himself would not have wished to remain alive, then the wishes of other family members, or politicians, religious leaders or the UNCRPD are not relevant. Treatment should be withdrawn.

If Vincent would have wished to remain alive, then artificial nutrition and hydration should potentially be continued. If there is disagreement about what the patient’s wishes were or would be, there is a need for an impartial process such as a court to weigh up the available evidence. In Lambert’s case, the Conseil d’Etat and the European Convention on Human Rights had carefully weighed up such evidence and found that it did not support continuation of treatment.^6^

Drawing on ethical dissensus, we should accept reasonable disagreement and provide treatment that respects the wishes of the patient. We recommend erring on the side of providing options that are of possible benefit or that are most likely to respect the prior autonomy of the patient.

## Is continuation of treatment an unreasonable use of limited medical resources?

However, there are limits to reasonable dissensus. Notably, where providing treatment options to one patient would negatively affect the options available to other patients, it may not be fair to simply defer to the wishes of the patient.

### In favour of withdrawal

There is an argument which is rarely made in relation to such cases (relevant exceptions are the studies by Kondziella *et al* and Daniels^9 22^). Even if Lambert had wished to continue to be treated, there is a reason not to do this: distributive justice. People do not have the right to demand limited community or medical resources such as artificial nutrition and hydration, nursing and hospital care. Such resources must be allocated according to reasonable objective values.

No matter how much a person wants to be kept alive in an unconscious state, we must ask: is this a fair and just use of community resources?

In the UK, it is estimated that the nursing home care for patients in a VS costs £90 000–120 000 per year.^23^ Wade has pointed out that this value is considerably in excess of the £20 000–30 000 cost/quality-adjusted life years (QALY) threshold commonly used for deliberations about providing novel therapies in the National Health Service (NHS).^24^ As an example, the NHS has recently declined to fund the novel therapy for cystic fibrosis lumacaftor/ivacaftor.^25^ This drug costs a similar amount per year per patient (£105 000) as the nursing home care for patients in VS; however, the benefit for patients with cystic fibrosis appears considerably greater (an additional 2.9 years of life/2.4 QALY on average).^26^ Although it may be considered controversial to assess whether nursing home care for VS is cost-effective, it does not seem controversial or even contestable that funding lumacaftor/ivacaftor would be *more cost-effective*.iii

From information available in the public domain, it does not appear that Lambert has other major health conditions (ie, affecting other organs than his brain). However, imagine that he developed renal failure and dialysis were judged to be consistent with his prior wishes. In contrast with artificial nutrition and hydration, there is widespread acceptance that renal dialysis should not be maintained for patients in VS. This is accepted, even in countries that are normally loathe to accept rationing of treatment. An American Society of Nephrology shared decision-making guideline indicates that dialysis should be withheld or withdrawn from ‘patients with irreversible, profound neurological impairment such that they lack signs of thought, sensation, purposeful behaviour and awareness of self and environment’.^27 28^ (Of relevance, the estimated annual cost of dialysis is approximately £30 000,^29^ one-third the cost of long-term nursing home care.) In the case of Tony *Bland*, artificial nutrition and hydration were classified as ‘medical’ treatments.^11^ This suggests that they should be treated in the same way as dialysis—and potentially withheld from patients in VS.

### Against withdrawal

Others would argue that it would be wrong to withdraw artificial nutrition and hydration from patients in a VS, if they would have wanted such treatment.

There is no question that the nursing and daily care of patients with severe disorders of consciousness is expensive. The cost of treatment is obviously higher than the cost-effectiveness limits that are sometimes used to decide about treatment.

However, many people do not accept that artificial nutrition and hydration are ‘treatment’,^30^ and would strongly oppose the idea that nursing home care should be regarded as optional. Some hold that provision of food and water is a basic human right and there is a moral obligation to provide it where it is possible to do so.

Moreover, decisions about allocation of resources are not made solely on the basis of maximising benefit. Other values are important to our community, and those values need to be balanced against benefit.^31 32^ For example, giving weight to fairness may mean departing from pure cost-effectiveness thresholds.^31^ In the case of patients with VS, fairness or equality would potentially favour providing treatment. After all, patients with less severe neurological impairment would unquestionably receive artificial nutrition and hydration if this were desired. It is deeply unfortunate and unfair for someone to sustain brain injury that leaves them permanently unconscious. It is arguably doubly unfair if they are then denied treatment (which they would have desired) because of the severity of their injury.^33^

Moreover, when society makes decisions about allocating treatment, it must assess whether there is a basic minimum level that should be provided to all patients. Some treatments may need to be rationed (ventilators, cancer drugs, organs for transplantation), but other treatments are provided uniformly where they offer some possible benefit (pain relief, basic nursing care). One justification for setting minimum standards, of this sort, is that this reflects a prioritarian concern for the worst off.^34^

Societies may differ in which treatments they regard as part of a basic level of healthcare provided to any and all patients, and which will be provided selectively. In the UK, for example, it would be generally judged to be appropriate not to admit to intensive care a patient in VS, nor to provide renal dialysis, nor to list for organ transplantation. However, for the present, at least, artificial nutrition and hydration and nursing home care are not subjected to rationing. That is, indeed, why it has never been subject to the sort of cost-effectiveness analysis that lumacaftor/ivacaftor has been.

We have suggested above that the UNCRPD should not classify as discrimination a decision to withhold treatment based on a patient’s wishes (and his interests). However, the committee may view differently an argument based on cost-effectiveness and disability. If that were the basis for stopping treatment in Lambert’s case, the UNCRPD could potentially have concluded that this breached his Article 25 right to health, and Article 2 right to life.

On the other hand, unless there is a reason to treat artificial nutrition and hydration differently from other treatments, it is difficult to see how this would not also apply to decisions to withhold dialysis, organ transplantation or intensive care admission.

### Dissensus

There is reasonable disagreement about how to allocate healthcare resources like long-term nursing home care. However, unlike questions of individual benefit/harm, this cannot be resolved by deferring to the preferences of the patient (or surrogate). Some people now demand intensive care even in the face of meeting criteria for death.^35^ And the end point of dementia, including Alzheimer’s disease, is unconsciousness. There will be large numbers of people whose lives could be prolonged who are unconscious.^36^ In the dissensus approach, we proposed that treatments that are *probably* of overall benefit and probably fall within the affordability limits of the healthcare system should be provided.^10^ There is then a question of what this means for continued treatment for patients in VS.

Our aim here is not to resolve the question of whether nursing home care and artificial nutrition and hydration should be withheld from patients in VS on the basis of limited resources. Instead, our aim is to point out that it is *that question* which needs to be addressed and resolved at a community level.

The focus of debate about treatment for patients with severe disorders of consciousness needs to shift away from the question of whether it is permissible to allow them to die—instead to the question of whether it is permissible to keep them alive indefinitely.^iv^ If they choose to use their own funds for this purpose, there is no obvious distributive justice objection.^v^ However, when they request the use of limited community resources, then such a decision becomes a communal one.

There is clearly going to remain disagreement about allocation of resources. The dissensus approach does not settle how such disagreement should be resolved. A democratic process of deliberation will necessarily tend to favour the values and views of the majority. However, deliberators should take into account the range of values and views held within society. There may not be a single right way to balance competing values in the face of reasonable disagreement. One option may be to settle on a fair process for allocation of resources.^22^

A community like France or the UK might decide that it will keep the unconscious alive with medical treatment like artificial nutrition and hydration, if they so desire. Well-resourced healthcare systems may be able to afford to provide this as a basic minimum level of healthcare. If they do, it may mean that nursing home care and community nursing should receive more funding that they do at present, at the expense of hospitals and pharmaceutical budgets (and, inevitably, reducing treatment availability for other patients).

However, that decision should be made in an open, transparent fully informed and rational way, with awareness of the opportunity cost that it involves. One estimate suggests that there are between 4000 and 16 000 patients with VS in nursing homes in the UK.^37^ At the same time, there are approximately 10 000 patients in the UK with cystic fibrosis.^38^

We will not all agree the right way to allocate limited resources, but given the large numbers of affected patients, and ongoing examples of conflict, these uncomfortable questions must be confronted.

## Footnotes

i These contrasting reactions are not mutually exclusive. Many people, including some family members, might experience both sentiments.^39^

ii We are grateful to a reviewer for highlighting this point.

iii There are methodological differences between the cost analysis that we have quoted for lumacaftor/ivacaftor and the cost estimate provided for nursing home care. For example, the price quoted for the former only includes the drug costs. Our aim is not to conduct a formal comparison of the cost-effectiveness of these two different interventions, but to point to the contrasting decisions that are made about interventions that might have a similar cost impact.

iv In the Court of Protection in the UK, decisions are already framed this way—focused on whether treatment is in the patient’s best interests (rather than whether it is in their best interests to die).^40^ Nevertheless, the wider international community debate (as the Lambert case illustrates) continues to focus on the permissibility of allowing to die.

v One possible objection is that self-funding treatment does not remove issues of collective harm. Given the shortage in the supply of carers, families who pay for the nursing home care of a family member will reduce the number of carers available to provide nursing care to other patients. We are grateful to a reviewer for highlighting this point.

## References

[1] BBC News. Vincent Lambert: life support must resume after court reverses ruling. 2019.

[2] Breeden A Hours after french patient is taken off life support, a court orders it be restored. New York Times 2019.

[3] Kishore RR, Lambert V Dignity in dying and the european court: a critical evaluation and the global reflections. 2016;23:141. 10.1163/15718093-12341381 27228683

[4] Veshi D Comments on the Lambert case: the rulings of the French Conseil d’État and the European Court of Human Rights. Med Health Care Philos 2017;20:187–93. 10.1007/s11019-016-9724-3 27581426

[5] Horn R The ’French exception': the right to continuous deep sedation at the end of life. J Med Ethics 2018;44:204–5. 10.1136/medethics-2017-104484 29056584 PMC5869460

[6] Case of Lambert and Others v. France. Application No. 46043/14, Judgment dated 5 June, 2015: ECHR, 2015.

[7] Jacquin J-B Affaire Vincent Lambert: la Cour de cassation autorise un nouvel arrêt des traitements. Le Monde 2019.

[8] Gipson J, Kahane G, Savulescu J Attitudes of Lay People to Withdrawal of Treatment in Brain Damaged Patients. Neuroethics 2014;7:1–9. 10.1007/s12152-012-9174-4 24600485 PMC3933752

[9] Kondziella D, Cheung MC, Dutta A Public perception of the vegetative state/unresponsive wakefulness syndrome: a crowdsourced study. PeerJ 2019;7:e6575 10.7717/peerj.6575 30863687 PMC6408911

[10] Wilkinson D, Savulescu D Ethics, conflict and medical treatment for children: from disagreement to dissensus: Elsevier, 2018. 30835404

[11] Airedale NHS Trust v Bland. 1993 [1993] AC 789.

[12] Mitchell KR, Kerridge IH, Lovat TJ Medical futility, treatment withdrawal and the persistent vegetative state. J Med Ethics 1993;19:71–6. 10.1136/jme.19.2.71 8331640 PMC1376191

[13] Code de la sante publique, Chapitre préliminaire: Droits de la personne. 2016 Article L1110-5- 1.

[14] Pullman D Death, dignity, and moral nonsense. J Palliat Care 2004;20:171–8. 10.1177/082585970402000309 15511036

[15] Allmark P Death with dignity. J Med Ethics 2002;28:255–7. 10.1136/jme.28.4.255 12161582 PMC1733631

[16] Wilkinson D Futility The international encyclopedia of ethics: Wiley, 2017.

[17] Macklin R Dignity is a useless concept. BMJ 2003;327:1419–20. 10.1136/bmj.327.7429.1419 14684633 PMC300789

[18] Killmister S Dignity: not such a useless concept. J Med Ethics 2010;36:160–4. 10.1136/jme.2009.031393 20211996

[19] Vatican News. Vincent Lambert: French court orders life support to resume. 2019.

[20] United Nations. United Nations convention on the rights of persons with disabilities. 2007. 10.1515/9783110208856.203 18348362

[21] Scholten M, Gather J Adverse consequences of article 12 of the UN Convention on the Rights of Persons with Disabilities for persons with mental disabilities and an alternative way forward. J Med Ethics 2018;44:226–33. 10.1136/medethics-2017-104414 29070707 PMC5869457

[22] Daniels N Just health: meeting health needs fairly. Cambridge: Cambridge University Press, 2008.

[23] Formby A, Cookson R, Halliday S Cost analysis of the legal declaratory relief requirement for withdrawing Clinically Assisted Nutrition and Hydration (CANH) from patients in the Permanent Vegetative State (PVS) in England and Wales. CHE Research Paper. York, UK: Centre for Health Economics, University of York, 2015.

[24] Wade DT Using best interests meetings for people in a prolonged disorder of consciousness to improve clinical and ethical management. J Med Ethics 2018;44:336–42. 10.1136/medethics-2017-104244 28912289

[25] Wise J NHS and Vertex remain deadlocked over price of cystic fibrosis drug. BMJ 2019;364:l1094 10.1136/bmj.l1094 30850360

[26] Dilokthornsakul P, Patidar M, Campbell JD Forecasting the Long-Term Clinical and Economic Outcomes of Lumacaftor/Ivacaftor in Cystic Fibrosis Patients with Homozygous phe508del Mutation. Value Health 2017;20:1329–35. 10.1016/j.jval.2017.06.014 29241892

[27] Patel SS, Holley JL Withholding and withdrawing dialysis in the intensive care unit: benefits derived from consulting the renal physicians association/american society of nephrology clinical practice guideline, shared decision-making in the appropriate initiation of and withdrawal from dialysis. Clin J Am Soc Nephrol 2008;3:587–93. 10.2215/CJN.04040907 18256375 PMC6631085

[28] Skold A, Lesandrini J, Gorbatkin S Ethics and health policy of dialyzing a patient in a persistent vegetative state. Clin J Am Soc Nephrol 2014;9:366–70. 10.2215/CJN.03410313 24115197 PMC3913231

[29] National Kidney Federation. Transplantation cost-effectiveness. 2010 Available: http://www.kidney.org.uk/archives/news-archive-2/campaigns-transplantation-trans-cost-effect/ (Accessed 27 Nov 2010).

[30] National Catholic Bioethics Center. Nutrition and hydration. The National Catholic Bioethics Center 2013 Available: https://www.ncbcenter.org/files/5314/4916/3492/NCBCsummFAQ_NutritionHydration.pdf (Accessed 08 Jul 2019).

[31] Wilkinson D, Savulescu J Prioritisation and parity: which disabled infants should be candidates for scarce life-saving treatment : Wasserman D, Cureton A, Oxford handbook of philosophy and disability: Oxford University Press, 2018.

[32] Norheim OF A note on Brock: prioritarianism, egalitarianism and the distribution of life years. J Med Ethics 2009;35:565–9. 10.1136/jme.2008.028845 19717696

[33] Harris J QALYfying the value of life. J Med Ethics 1987;13:117–23. 10.1136/jme.13.3.117 3669036 PMC1375658

[34] Parfit D Equality and Priority. Ratio 1997;10:202–21. 10.1111/1467-9329.00041

[35] Luce JM The uncommon case of Jahi McMath. Chest 2015;147:1144–51. 10.1378/chest.14-2227 25846530

[36] Cuff A Dementia - a global epidemic. BioMedCentral 2015 Available: https://blogs.biomedcentral.com/on-medicine/2015/05/18/dementia-global-epidemic/ [Accessed 10 Jun].

[37] Parliamentary Office of Science and Technology. POSTnote: Vegetative and Minimally Conscious States. Houses of Parliament. 2015 Available: https://researchbriefings.parliament.uk/ResearchBriefing/Summary/POST-PN-489 (Accessed 10 Jun 2019).

[38] Cystic fibrosis FAQs. Cystic Fibrosis trust. Available: https://www.cysticfibrosis.org.uk/what-is-cystic-fibrosis/faqs

[39] Kitzinger C, Kitzinger J Withdrawing artificial nutrition and hydration from minimally conscious and vegetative patients: family perspectives. J Med Ethics 2015;41:157–60. 10.1136/medethics-2013-101799 24425753 PMC4316914

[40] British Medical Association, Royal College of Physicians. Clinically-assisted nutrition and hydration (CANH) and adults who lack the capacity to consent: Guidance for decision-making in England and Wales. 2018 Available https://www.bma.org.uk/advice/employment/ethics/mental-capacity/clinically-assisted-nutrition-and-hydration/clinically-assisted-nutrition-and-hydration-canh-guidance (Accessed 08 Jul 2019).

